# Descriptive analysis of adverse drug reaction reports in children and adolescents from Germany: frequently reported reactions and suspected drugs

**DOI:** 10.1186/s40360-021-00520-y

**Published:** 2021-10-07

**Authors:** Diana Dubrall, Sarah Leitzen, Irmgard Toni, Julia Stingl, M. Schulz, Matthias Schmid, Antje Neubert, Bernhardt Sachs

**Affiliations:** 1 Institute for Medical Biometry, Informatics and Epidemiology, University Hospital of Bonn, Bonn, Germany; 2 Research Division, Federal Institute for Drugs and Medical Devices, Bonn, Germany; 3 Department of Physics, Chemistry and Pharmacy, University of Southern Denmark, Odense, Denmark; 4 Department of Paediatrics and Adolescent Medicine, University Hospital Erlangen, Erlangen, Germany; 5 Institute of Clinical Pharmacology, University Hospital of the RWTH Aachen, Aachen, Germany; 6 Central Research Institute of Ambulatory Health Care in Germany, Berlin, Germany; 7 Department for Dermatology and Allergy, University Hospital Aachen, Aachen, Germany

**Keywords:** Adverse drug reactions, Adverse drug reaction reports, Adverse drug reaction database analysis, Children, Adolescent, Off-label use, Side effects

## Abstract

**Background:**

Adverse drug reactions (ADRs) in the pediatric population may differ in types and frequencies compared to other populations. Respective studies analyzing ADR reports referring to children have already been performed for certain countries. However, differences in drug prescriptions, among others, complicate the transferability of the results from other countries to Germany or were rarely considered. Hence, the first aim of our study was to analyze the drugs and ADRs reported most frequently in ADR reports from Germany referring to children contained in the European ADR database (EudraVigilance). The second aim was to set the number of ADR reports in relation to the number of drug prescriptions. These were provided by the Research Institute for Ambulatory Health Care in Germany.

**Methods:**

For patients aged 0–17 years 20,854 spontaneous ADR reports were received between 01/01/2000–28/2/2019. The drugs and ADRs reported most frequently were identified. Stratified analyses with regard to age, sex and drugs used “off-label” were performed. Reporting rates (number of ADR reports/number of drug prescriptions) were calculated.

**Results:**

Methylphenidate (5.5%), ibuprofen (2.3%), and palivizumab (2.0%) were most frequently reported as suspected. If related to the number of drug prescriptions, the ranking changed (palivizumab, methylphenidate, ibuprofen). Irrespective of the applied drugs, vomiting (5.4%), urticaria (4.6%) and dyspnea (4.2%) were the ADRs reported most frequently. For children aged 0–1 year, drugs for the treatment of nervous system disorders and foetal exposure during pregnancy were most commonly reported. In contrast, methylphenidate ranked first in children older than 6 years and referred 3.5 times more often to males compared to females. If age- and sex-specific exposure was considered, more ADR reports for methylphenidate referred to children 4–6 years and females 13–17 years. Drugs for the treatment of nervous system disorders ranked first among “off-label” ADR reports.

**Conclusions:**

Our analysis underlines the importance of putting the number of ADR reports of a drug in context with its prescriptions. Additionally, differences in age- and sex-stratified analysis were observed which may be associated with age- and sex-specific diseases and, thus, drug exposure. The drugs most frequently included in “off-label” ADR reports differed from those most often used according to literature.

**Supplementary Information:**

The online version contains supplementary material available at 10.1186/s40360-021-00520-y.

## Background

Children and adolescents are often not included in clinical trials, hence, data with regard to the efficacy and safety of drug therapy in children and adolescent are lacking [1]. Thus, many medicinal products are used off-label in children [2, 3], i.e. outside their approval conditions (e.g. authorized age) [4, 5]. ADRs associated with drugs used in the pediatric population need, however, specific evaluation as they may substantially differ - in terms of frequency, nature and severity - from those occurring in adults [6]. Differences with regard to the pharmacokinetics among other factors may account for this observation [3].

In Germany, 1.7% of children taking medication on an *outpatient* basis experience at least one ADR [4], whereas 10.0% of all pediatric *inpatients* are estimated to develop an ADR [4]. The incidence of ADRs, leading to hospital admission, is appreciated to be 1% [7] for children in Germany.

With regard to the drugs most often used in German children and adolescents (3–17 years), drugs for the treatment of respiratory disorders ranked first, followed by varia and drugs for the treatment of musculoskeletal and connective tissue disorders [8]. In contrast, cardiovascular drugs, antineoplastic agents and drugs for the treatment of sensory organs were most often used off-label [9].

Concerning drug related ADRs, antiinfectives and antiepileptics were most frequently associated in *hospitalized* children or children admitted *to the hospital* in a systematic review [10]. In outpatient children, non-steroidal anti-inflammatory drugs (NSAID), besides antiinfectives, were most frequently associated with ADRs [10]. Regarding ADRs, these most frequently referred to the system organ classes “general disorders and administration site conditions” (31%), “skin and subcutaneous tissue disorders” (18%) and “nervous system disorders” (15%) in a Danish ADR database study [11].

Several studies already investigated ADRs in children using ADR databases. However, these studies referred to particular countries or regions (Denmark [11], Sweden [12, 13], Brazil [14], Malaysia [15], Korea [16], the US [17], EU [18]) and, thus, may differ from the ADR reports referring to children and adolescents in Germany. Differences in (i) reporting obligations and behaviors, (ii) health care systems, or (iii) study designs (e.g. inclusion or exclusion of ADR reports related to vaccines [11, 13, 17, 18]) may lead to substantial deviations. Additionally, the number of ADR reports may be influenced by the number of drug prescriptions which was rarely taken into account in these studies. Likewise, ADR reports referring to drugs used off-label may also differ between countries due to differences in off-label prescribing, national guidelines and marketing authorizations. Further on, the reported drugs and ADRs may differ compared to ADR reports not associated with “off-label use”. Finally, one general limitation of such ADR database analyses is the unknown amount of underreporting.

The first aim of the presented analysis was to investigate the drugs and ADRs most often involved in ADR reports referring to children and adolescents from Germany. Since drug prescriptions may differ depending on age and sex we additionally performed age- and sex-stratified analysis.

Secondly, the number of ADR reports was set in relation to the number of drug prescriptions in order to evaluate to what extent drug exposure may impacts on the evaluation of the most frequently reported drugs in our analysis.

As a third aim, the drugs and ADRs most often mentioned in ADR reports associated with off-label use, were examined in order to investigate if these differ from those ADR reports not associated with off-label use.

## Methods

### ADRs

An ADR is defined as a response to a medicinal product normally used in patients which is noxious and unintended [19]. Since 2012 the ADR definition was widened and now also covers ADRs that occurred with an overdose, off-label use, misuse, abuse or medication error [20].

A more detailed description about reporting obligations and reporting channels can be found elsewhere [1, 21, 22].

### EudraVigilance

All ADR reports from the European Economic Area (EEA) are stored in the European ADR database EudraVigilance of the European Medicines Agency (EMA) [23]. EVDAS is the data analysis system of EudraVigilance. Public access to EudraVigilance is granted, although different levels of access apply to different stakeholders [24].

In EudraVigilance, ADRs and the patient’s history are coded in accordance with MedDRA terminology [25] and drugs with the EudraVigilance medicinal product dictionary (XEVMPD or Article 57 database) [26]. MedDRA terminology has a hierarchical structure not only coding ADRs but also diagnoses, investigations or product use issues such as off-label use. ADRs can be analysed on a summarizing level, the system organ class (SOC) level, which describes the organ system in which the ADR occurs, or on more detailed levels such as the preferred term (PT) level, which codes among others the reported symptoms.

### Identification of cases in EVDAS

**Fig. 1:**
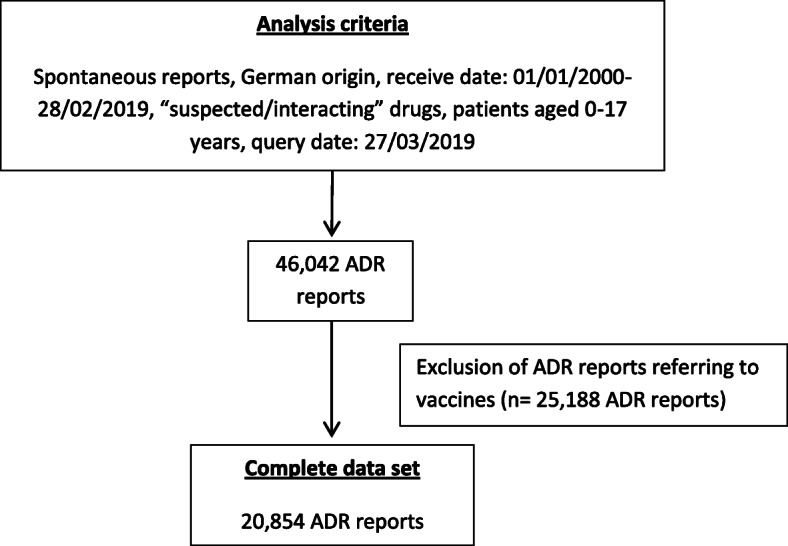
Identification of ADR reports referring to children and adolescents from Germany

We identified all spontaneous ADR reports of suspected/interacting drugs for German children and adolescents (0–17 years) (in the following “children”) received between 01/01/2000–28/02/2019 (*n* = 46,042). Roughly half of the ADR reports referred to vaccines. Vaccination is a process different from drug administration and may influence age- and sex-stratified analysis through age specific vaccination schedules. Thus, ADR reports referring to vaccines were excluded just as in other studies [14, 16, 17]. Finally, our dataset consisted of 20,854 spontaneous ADR reports (in the following “ADR reports”). These ADR reports informed about 61,824 ADRs (PT-level; definition see strategy of analyses) and 31,669 suspected/interacting drugs or drug combinations (Fig. 1).

Further information about the number of ADR reports for German children in relation to the number of children in the German population and assumed drug-exposed children as well as the reported seriousness criteria and reporting sources can be found in a separate publication by our group [27].

### Strategy of analyses

The final dataset was analyzed with regard to (i) the demographical parameters, (ii) the patient histories, (iii) the ADRs and drugs reported most frequently, (iv) the ADRs, demographical parameters as well the proportion of serious ADR reports for the most frequently reported drugs and (v) the number of ADR reports per administration route. Means and medians were calculated for the patients’ age, and frequency distributions for all other results. In each case the five most frequently reported results are presented.

In accordance with the legal definition an ADR is classified as serious if it was life-threatening, led to death, hospitalisation or prolonged hospitalisation, disabilities or congenital anomalies [20].

Concerning the ADRs reported most frequently, one analysis on the summarizing SOC-level and one on the more detailed PT-level of the MedDRA terminology [25] was performed. The PT-level was also applied for the analysis of the patient’s history.

The reported drugs were analysed on the drug substance level representing single drug substances and their combination products (if available).

Age- and sex-stratified analysis were performed. Therefore, a modified age stratification according to the National Association of Statutory Health Insurance Physicians [28] was used (“0–1 month”, “2 months-1 year”, “2–3 years”, “4–6 years”, “7–12 years” and “13–17 years”). In both, the three drugs with their three ADRs most frequently reported are presented.

To detect sex-related ADR differences, Odds Ratios (OR) with Bonferroni adjusted confidence intervals (CI) for the most frequently reported ADRs for females and males overall ages were calculated. Sex-specific ADRs (ADRs which only occur in one sex (e.g. uterus-related events in women [25])) were not excluded. Presented are the ten most frequently reported ADRs for males and females.

Since ADR reports of drugs used off-label may differ in types of drugs and ADRs reported, we analyzed ADR reports referring to “off-label use” separately. Therefore, we identified the number of ADR reports referring to “off-label use” by summarizing appropriate PTs of the MedDRA terminology [25] (see Supplementary file 1). It has to be considered that not all ADR reports might be coded with one of these PTs, and, thus, may not be identified. In order to address this issue, we additionally examined the age of the patients in the ADR reports of the ten most frequently reported drugs and compared it to the indicated age mentioned in the official product information. Unfortunately, the identified ADR reports mostly referred to children aged 0–1 years, hence, most likely representing ADRs which occurred in context with drug exposure during pregnancy or breastfeeding. After exclusion of these ADR reports, ADR reports referring to “off-label use” per authorized age remained only for atomoxetine, methylphenidate and risperidone.

### Calculation of reporting rates

The number of drug prescriptions for the five most frequently reported drugs overall and in the age- and sex-stratified analyses were provided by the Research Institute for Ambulatory Health Care in Germany [29]. Drug prescription data were only available since 01/01/2009 and include all drugs prescribed for outpatients. Children younger than 2 months were excluded by technical reasons (drug prescriptions referenced to the mother until the child had its own individual insurance number). For the calculation of reporting rates the number of ADR reports for the most frequently reported drugs in the whole dataset had to be aligned to the time period 01/01/2009–28/02/2019, at first. Then, ratios were calculated using the identified number of ADR reports divided by (i) the number of drug prescriptions (including patients with more than one drug prescription) and (ii) the number of drug prescriptions related to different patients (drug-exposed patients). The results are presented as the number of ADR reports per 100,000 drug prescriptions/drug prescriptions for different patients (= reporting rates).

### Sensitivity analysis

All of the analyses were conducted computer-based. No evaluation of each individual case with regard to the causal relationship and the quality of the ADR reports is performed by default. Thus, we assessed a random sample of 100 ADR reports (0.5% (100/20,854)) with regard to their quality (=completeness) and the causal association between the reported ADRs and the applied drugs. For the assessment of the documentation quality a published World Health Organization (WHO) score (vigiGrade) was applied [30]. The calculation of the score was modified as it was evaluated for the leading ADR, only [30]. The calculated completeness score was 0.6 [0.6–0.7] (per definition: > 0.8 = „well-documented“). Most of the incomplete data referred to the variable *time to onset* (TTO) (51.0%).

For the assessment of the causal association the WHO-criteria were used [31]. In our sample, 77.0% of the ADR reports had an at least possible causal relationship (1.0% “certain” + 5.0% “probable/likely” + 71.0% “possible”).

## Results

### Analysis of the complete data set (*n* = 20,854 ADR reports)

The mean age of the patients was 8.5 years and slightly more ADR reports referred to males than to females (51.2% vs. 44.9%) (Table 1).

**Table 1:** Analysis of the characteristics reported most frequently in the complete data set

	Complete data set (*n* = 20,854)
*Patients demographics*	
mean age (+/− standard deviation) [years]	8.5 (+/− 6.1)
median age (interquartile range) [years]	9.0 (2.0–14.0)
female	44.9% (*n* = 9354)
male	51.2% (*n* = 10,670)
unknown	4.0% (*n* = 830)
*The five patient histories most frequently reported* ^*a*^	
cases with information	47.7% (*n* = 9942)
1.	asthma (6.8%; 672/9942)
2.	premature baby (5.3%; 522/9942)
3.	rhinitis allergic (4.8%; 479/9942)
4.	seasonal allergy (4.7%; 469/9942)
5.	epilepsy (4.0%; 394/9942)
*The five ADRs most frequently reported (SOC-level)* ^*a*^	
1.	general disorders and administration site conditions (24.1%; *n* = 5030)
2.	nervous system disorders (20.6%; *n* = 4290)
3.	injury, poisoning and procedural complications (18.2%; *n* = 3785)
4.	gastrointestinal disorders (17.9%; *n* = 3733)
5.	skin and subcutaneous tissue disorders (17.2%; *n* = 3582)
*The five ADRs most frequently reported (PT-level)* ^*a*^	
1.	vomiting (5.4%; *n* = 1125)
2.	urticaria (4.6%; *n* = 954)
3.	dyspnoea (4.2%; *n* = 883)
4.	nausea (3.9%; *n* = 803)
5.	rash (3.1%; *n* = 637)
*The five drug substances reported most frequently* ^*a*^	
1.	methylphenidate (5.5%; *n* = 1151)
2.	ibuprofen (2.3%; *n* = 470)
3.	palivizumab (2.0%; *n* = 411)
4.	atomoxetine (2.0%; *n* = 407)
5.	etanercept (1.9%; *n* = 389)
*The five application routes most often reported irrespective of the applied drug substance/substances* ^*b*^	
1.	oral (41.9%; *n* = 8739)
2.	subcutaneous (11.0%; *n* = 2299)
3.	transplacental (10.5%; *n* = 2181)
4.	intravenous (8.0%; *n* = 1659)
5.	intramuscular (2.0%; *n* = 427)

^a^presented are the five most frequently reported patient histories, ADRs and drug substances. Patients’ histories were analysed on the PT-level and ADRs on the PT- and the SOC-level of the MedDRA terminology [25]. Single drug substances and their combination products (if available) were summarized. One ADR report may contain information about more than one (i) pre-existing disease, (ii) ADR or (iii) drug substance, therefore, the number of reported patient histories, ADRs or drug substances may exceed that of the ADR reports

^b^presented are the five application routes most frequently reported irrespective of the applied drug substances and the number of applied drugs per ADR report. Multiple assignments are possible if one ADR report contains more than one drug referring to different application routes. 24.4% and 8.2% of the ADR reports contained drugs for which the application route was designated as not available or unknown

Table 1 presents patients demographics and histories, the ADRs on SOC- and PT-level of the MedDRA terminology, the drugs substances and the application routes most frequently reported in the complete data set

Roughly 47.7% (9942/20,854) of all ADR reports provided information about the medical history of the patient. In these ADR reports the most frequently reported pre-existing conditions were asthma 6.8% (672/ 9942), premature baby 5.3% (522/9942) and rhinitis allergic 4.8% (479/9942).

On a more summarizing analysis level (SOC-level), irrespective of the reported suspected drug substances, the ADRs reported most frequently referred to the SOC “general disorders and administration site conditions” (24.1%), followed by “nervous system disorders” (20.7%), and “injury, poisoning and procedural complications” (18.1%). “Vomiting” (5.4%), “urticaria” (4.6%), “dyspnoea” (4.2%), “nausea” (3.9%), and “rash” (3.1%) were the ADRs most frequently reported on the more detailed analysis level (PT-level).

Over the complete data set the five drug substances most frequently reported as suspected were methylphenidate (5.5%), ibuprofen (2.3%), palivizumab (2.0%), atomoxetine (2.0%) and etanercept (1.9%). Differences were observed in mean age, male/female ratio and the proportion of serious ADR reports between these drugs (Table 2). Methylphendiate and atomoxetine were roughly 3.5 and 4.2 times more often reported for males than for females with a higher mean age of the patients compared to ibuprofen and palivizumab. The proportion of serious ADR reports was lowest for etanercept and highest for palivizumab. With regard to the ADRs most frequently reported, a more drug-specific ADR profile emerged. In this regard, “headache” (5.3%), “decreased appetite” (4.7%) and “tachycardia” (4.2%) were reported most often with methylphenidate. “Vomiting” (10.4%) was reported most often with ibuprofen (if “suicide attempt” and “intentional overdose” are not considered as an ADR) and “respiratory syncytial virus infection” (40.1%) with palivizumab. An individual case assessment confirmed that most of the cases reported for palivizumab were related to a lack of efficacy. “Suicide attempt” (13.8%) and “suicidal ideation” (14.0%) were the most common reported PTs for ibuprofen and atomoxetine.

**Table 2:** Drug-stratified analysis

Drug substances	Methylphenidate ^a^ (5.5%; *n* = 1151)	Ibuprofen ^a^ (2.3%; *n* = 470)	Palivizumab ^a,b^ (2.0%; *n* = 411)	Atomoxetine ^a^ (2.0%; *n* = 407)	Etanercept ^a^ (1.9%; *n* = 389)
Patients demographics					
Mean age (+/− standard deviation) [years]	11.2 (+/−3.4)	7.9 (+/−6.0)	0.2 (+/− 0.5)	11.4 (+/− 3.1)	11.4 (+/− 4.5)
Median age (interquartile range) [years]	11.0 (9.0–14.0)	7.0 (2.0–14.0)	0.0 (0.0–0.0)	12.0 (9.0–14.0)	12.0 (9.0–15.0)
Male/female ratio	3.5:1.0	1.0:1.0	1.3:1.0	4.2:1.0	0.5:1.0
Serious	77.5%	76.6%	96.4%	87.7%	39.6%
ADRs most frequently reported					
1.	headache (5.3%; *n* = 61)	suicide attempt (13.8%; n = 65)	respiratory syncytial virus infection (40.1%; n = 165)	suicidal ideation (14.0%; n = 57)	injection site pain (16.7%; *n* = 65)
2.	decreased appetite (4.7%; *n* = 54)	intentional overdose (11.3%; n = 53)	respiratory syncytial virus bronchiolitis (16.1%; n = 66)	aggression (12.5%; *n* = 51)	condition aggravated (6.9%; n = 27)
3.	tachycardia (4.2%; n = 48)	vomiting (10.4%; n = 49)	bronchitis (8.5%; n = 35)	nausea (7.9%; n = 32)	injection site erythema (5.1%; n = 20)
4.	tic (4.0%; n = 46)	urticaria (8.3%; n = 39)	pneumonia (7.3%; n = 30)	tachycardia (6.1%; *n* = 25)	drug ineffective (4.6%; n = 18)
5.	leukopenia (3.6%; n = 41)	nausea (6.4%; *n* = 6.4%)	respiratory failure (6.8%; n = 28)	fatigue (5.4%; n = 22)	nasopharyngitis (4.6%; *n* = 18)

^a^single drug substances and their combination products (if available) were summarized

^b^ADR reports for palivizumab were mostly related to a lack of efficacy

Table 2 presents the patients’ demographics and ADRs reported most frequently for the five drugs reported most frequently in the complete data set

Irrespective of the reported drug substances and the number of applied drugs per ADR report, the application route was oral in 41.9%, subcutaneous in 11.0% and transplacental in 10.5% of the ADR reports (Table 1).

### Reporting rates per 100,000 drug prescriptions or drug prescriptions for different patients

In relation to the specific exposure the ranking of the five drug substances reported most frequently in the complete data set (01/01/2000–28/2/2019) changed (Table 3). The reporting rate per 100,000 drug prescriptions or drug prescriptions for different patients was highest for etanercept, followed by palivizumab, atomoxetine, methylphenidate and ibuprofen.

**Table 3:** Reporting rates per 100,000 drug prescriptions/drug prescriptions for different patients

The five drug substances reported most frequently between 01/01/2000–28/2/2019* (number of ADR reports between 01/01/2009–28/02/2019) ^a^	Reporting rate per 100,000 drug prescriptions ^b^	Reporting rate per 100,000 drug prescriptions for different patients ^b^
Etanercept (*n* = 302)	546.6	4946.8
Palivizumab (*n* = 325) ^c^	86.3	446.1
Atomoxetine (*n* = 178)	19.8	229.5
Methylphenidate (*n* = 604)	5.8	85.4
Ibuprofen (*n* = 339)	0.6	2.2

^a^presented are the five drug substances most frequently reported. Single drug substances and their combination products (if available) were summarized

^b^calculation of reporting rates: number of ADR reports/number of drug prescriptions or number of ADR reports/number of drug prescriptions for different patients)

^c^ADR reports for palivizumab were mostly related to a lack of efficacy

Table 3 presents the ranking of the reporting rates for the five drug substances most frequently reported in the complete data set (see Table 1). The reporting rate was calculated for the time period 01/01/2009–28/02/2019, since substance-specific exposure data were only available for this time

### Age-stratified analysis of drugs and ADRs most frequently reported

Except for the age group 13–17 years, more ADR reports referred to males than to females (Table 4). The ranking of the drug substances most frequently reported as suspected differed depending on age. For instance, the five drugs reported most frequently in children 0–1 month are indicated for nervous system disorders. Likewise, three out of five drugs in children 2 months-1 year referred to this drug class. In contrast, ibuprofen and amoxicillin were the drug substances most frequently reported in children aged 2–3 years. Ibuprofen was also leading for children aged 4–6 years followed by methylphenidate. Methylphenidate ranked first in children 7–17 years.

**Table 4:** Age-stratified analysis of demographical parameters and the five drug substances and ADRs reported most frequently

Age groups	0–1 month (*n* = 2451)	2 month-1 year (*n* = 2302)	2–3 years (*n* = 1537)	4–6 years (*n* = 1929)	7–12 years (*n* = 5384)	13–17 years (*n* = 7251)
*Demographical parameters*						
female	40.2% (*n* = 986)	39.0% (*n* = 898)	43.5% (*n* = 669)	40.8% (*n* = 787)	37.9% (*n* = 2042)	54.8% (*n* = 3972)
male	54.2% (*n* = 1329)	52.6% (*n* = 1212)	50.4% (*n* = 775)	54.5% (*n* = 1049)	59.2% (*n* = 3186)	43.0% (*n* = 3119)
unknown	5.5% (*n* = 136)	8.3% (n = 192)	6.1% (*n* = 93)	4.8% (n = 93)	2.9% (*n* = 156)	2.2% (*n* = 160)
*The five drug substances most frequently reported as suspected in the respective age group* ^*a*^						
1.	venlafaxine (6.2%; *n* = 152)	palivizumab ^b^ (16.3%; *n* = 376)	ibuprofen (3.7%; *n* = 57)	ibuprofen (3.7%; *n* = 71)	methylphenidate (11.6%; *n* = 625)	methylphenidate (6.1%; *n* = 440)
2.	lamotrigine (5.5%; *n* = 134)	levetiracetam (2.9%; n = 66)	amoxicillin (3.1%; *n* = 48)	methylphenidate (3.3%; *n* = 64)	atomoxetine (4.5%; *n* = 241)	isotretinoin (2.8%; *n* = 204)
3.	citalopram (5.1%; *n* = 124)	ibuprofen (2.3%; *n* = 52)	cefaclor (3.0%; *n* = 46)	valproinic acid (2.7%; *n* = 53)	etanercept (2.6%; *n* = 138)	etanercept (2.6%; *n* = 190)
4.	quetiapine (4.2%; *n* = 104)	valproinic acid (2.0%; *n* = 45)	salbutamol (2.9%; *n* = 45)	oxcarbazepine (2.3%; n = 44)	insulin aspartate (1.8%; *n* = 96)	dienogest (2.6%; *n* = 187)
5.	sertraline (4.0%; n = 98)	topiramate (1.9%; n = 44)	valproinic acid (2.8%; *n* = 43)	amoxicillin (2.2%; *n* = 43)	ibuprofen (1.8%; n = 96)	levonorgestrel (2.6%; *n* = 185)
*The five ADRs most frequently reported in the respective age group* ^*a*^						
1.	foetal exposure during pregnancy (16.3%; *n* = 400)	foetal exposure during pregnancy (6.8%; *n* = 156)	vomiting (9.3%, *n* = 143)	vomiting (8.0%; *n* = 155)	urticaria (6.6%; *n* = 358)	suicide attempt (6.7%; *n* = 487)
2.	atrial septal defect (14.4%; *n* = 352)	respiratory syncytial virus infection (6.6%; *n* = 151)	accidental exposure to product by child (8.3%; n = 127)	urticaria (6.6%; *n* = 127)	dyspnoea (6.2%; *n* = 334)	nausea (5.8; *n* = 419)
3.	premature baby (12.2%; *n* = 298)	vomiting (5.7%; *n* = 131)	accidental overdose (5.9%; *n* = 91)	pyrexia (5.0%; *n* = 97)	vomiting (5.5%; *n* = 296)	dyspnoea(5.4%; *n* = 395)
4.	small for dates baby (9.7%; *n* = 238)	accidental exposure to product by child (4.1%; n = 95)	pyrexia (5.3%; *n* = 82)	rash (4.9%; *n* = 95)	nausea (5.2%; *n* = 279)	intentional overdose (5.3%; *n* = 387)
5.	neonatal respiratory distress syndrome (6.9%; *n* = 169)	pyrexia (4.1%; *n* = 94)	urticaria (5.3%; n = 82)	pruritus (4.7%; *n* = 91)	anaphylactic reaction (4.4%; *n* = 239)	vomiting (5.1%; *n* = 368)

^a^ presented are the five drugs substances and the five ADRs (PT-level) most frequently reported. Single drug substances and their combination products (if available) were summarized. One ADR report may contain information about more than one drug substance and more than one ADR, therefore, the numbers of reported drug substances and ADRs may exceed that of the ADR reports

^b^ ADR reports for palivizumab were mostly related to a lack of efficacy

Table 4 presents the demographical parameters, the drug substances and ADRs most frequently reported in the age-stratified analysis of the complete data set

Irrespective of the applied drug, “foetal exposure during pregnancy” was the ADR most often reported in children 0–1 years. In children 2–3 years “vomiting” ranked first followed by “accidental exposure to product by child” and “accidental overdose”. “Vomiting” remained the most frequently reported ADR in children aged 4–6 years, whereas “urticaria” was the ADR most often reported in children 7–12 years. Remarkably, “suicide attempt” was the condition reported mainly in children 13–17 years.

### Age- and sex-stratified analysis of drugs and their ADRs most frequently reported

In age- and sex-stratified analysis, no or only small differences regarding the drug substances and their ADRs most frequently reported were observed for males and females aged 0–3 years (Table 5). However, differences could be observed for males and females above 0–3 years, especially with regard to methylphenidate. Methylphenidate was the drug substance reported most frequently for males 4–17 years. In contrast, for females ibuprofen (4–6 years), methylphenidate (7–12 years) and dienogest including combination products (13–17 years) were the drug substances reported most frequently. However, the absolute number of ADR reports for methylphenidate referring to males 7–12 years (*n* = 490) was 4.2 times higher compared to females (*n* = 118).

**Table 5:** Age- and sex-stratified analysis of drug substances and their ADRs most frequently reported

Age groups	Females: rank of drug substances ^a^	Females: the three drug substances reported most frequently as suspected and their three ADRs reported most frequently in brackets	Males: rank of drug substances ^a^	Males: the three drug substances reported most frequently reported and their three ADRs reported most frequently in brackets
0–1 month (*n* = 2451)	1. venlafaxine (5.7%; *n* = 56)	atrial septal defect (32.1%; n = 18) neonatal respiratory distress syndrome (17.9%; n = 10) patent ductus arteriosus (16.1%; n = 9) ventricular septal defect (16.1%; n = 9)	1. venlafaxine (6.9%; *n* = 92)	atrial septal defect (20.7%; n = 19) neonatal respiratory distress syndrome (18.5%; *n* = 17) drug withdrawal syndrome neonatal (14.1%; n = 13) small for dates baby (14.1%; n = 13)
	2. lamotrigine (5.6%; *n* = 55)	atrial septal defect (40.0%; *n* = 22) foetal exposure during pregnancy (20.0%; *n* = 11) selective eating disorder (16.4%; n = 9)	2. lamotrigine (5.4%; *n* = 72)	atrial septal defect (18.1%; n = 13) foetal exposure during pregnancy (16.7%; n = 12) neonatal respiratory distress syndrome (9.7%; n = 7)
	3. citalopram (5.3%; *n* = 52)	foetal exposure during pregnancy (26.9%; n = 14) atrial septal defect (25.0%; n = 13) small for dates baby (19.2%; n = 10) ventricular septal defect (19.2%; n = 10)	2. citalopram (5.4%; n = 72)	foetal exposure during pregnancy (37.5%; *n* = 27) atrial septal defect (19.4%; n = 14) small for dates baby (19.4%; n = 14)
2 month-1 year (*n* = 2302)	1. palivizumab ^b^ (16.6%; *n* = 149)	respiratory syncytial virus infection (38.3%; *n* = 57) respiratory syncytial virus bronchiolitis (14.8%; *n* = 22) bronchitis (10.7%; *n* = 16)	1. palivizumab ^b^ (17.0%; *n* = 206)	respiratory syncytial virus infection (38.8%; *n* = 80) respiratory syncytial virus bronchiolitis (18.0%; *n* = 37) bronchitis (8.7%; *n* = 18)
	2. levetiracetam (3.5%; *n* = 31)	foetal exposure during pregnancy (51.6%; n = 16) premature baby (19.4%; *n* = 6) small for dates baby (16.1%; n = 5)	2. levetiracetam (2.6%; *n* = 32)	foetal exposure during pregnancy (53.1%; *n* = 17) atrial septal defect (15.6%; n = 5) exposure during pregnancy (12.5%; *n* = 4) premature baby (12.5%; n = 4),
	3. topiramate (2.3%; *n* = 21)	off-label use (19.0%; n = 4) seizure (14.3%; n = 3)	3. octocog alfa (2.5%; *n* = 30)	factor VIII inhibition (70.0%; *n* = 21), anti factor VIII antibody positive (23.3%; n = 7)
2–3 years (*n* = 1537)	1. amoxicillin (3.9%; *n* = 26)	tooth discolouration (26.7%; n = 7) rash (11.5%; n = 3)	1. ibuprofen (4.5%; *n* = 35)	urticaria (20.0%; n = 7) crying (11.4%; n = 4) angioedema (8.6%; n = 3), diarrhoea (8.6%; n = 3) eyelid oedema (8.6%; n = 3) lip swelling (8.6%; n = 3) vomiting (8.6%; n = 3)
	2. valproinic acid (3.4%; n = 23)	seizure (17.4%; n = 4) electroencephalogram abnormal (13.0%; n = 3) fatigue (13.0%; n = 3) liver disorder (13.0%; n = 3)	2. amoxicillin (4.0%; n = 31)	rash (19.4%; n = 6) vomiting (16.1%; n = 5) dizziness (9.7%; n = 3) urticaria (9.7%; n = 3)
	3. cefaclor (3.1%; n = 21)	tooth discolouration (19.0%; n = 4) urticaria (19.0%; n = 4) confusional state (14.3%; n = 3) joint swelling (14.3%; n = 3) vomiting (14.3%; n = 3)	3. cefaclor (3.1%; n = 24)	rash (16.7%; n = 4) urticaria (16.7%; n = 4) dyspnoea (12.5%; n = 3) erythema (12.5%; n = 3) pruritus (12.5%; n = 3) vomiting (12.5%; n = 3)
4–6 years (*n* = 1929)	1. ibuprofen (3.6%; *n* = 28)	urticaria (21.4%; n = 6) nausea (14.3%; n = 4) vomiting (14.3%; n = 4)	1. methylphenidate (5.0%; n = 52)	blood creatine phosphokinase increased (13.5%; n = 7) leukopenia (9.6%; n = 5) weight decreased (7.7%; n = 4)
	2. valproinic acid (3.3%; n = 26)	condition aggravated (15.4%; n = 4) blood creatinine phosphokinase increased (11.5%; n = 3) decreased level of consciousness (11.5%; n = 3) dystonia (11.5%; n = 3) encephalopathy (11.5%; n = 3) epilepsy (11.5%; n = 3) hyperthermia (11.5%; n = 3) movement disorder (11.5%; n = 3) myoclonus (11.5%; n = 3)	2. ibuprofen (4.1%; *n* = 43)	swelling face (16.3%; n = 7) urticaria (16.3%; n = 7) angioedema (11.6%; n = 5) eyelid oedema (11.6%; n = 5)
	3. oxcarbazepine (2.5%; n = 20)	hyponatraemia (35.0%; n = 7) fatigue (15.0%; n = 3)	3. montelukast (2.7%; n = 28)	aggression (25.0%; n = 7) seizure (14.3%; n = 4) abdominal pain (10.7%; n = 3) hallucination (10.7%; n = 3) nightmare (10.7%; n = 3) pyrexia (10.7%; n = 3) restlessness (10.7%; n = 3) vomiting (10.7%; n = 3)
7–12 years (*n* = 5384)	1. methylphenidate (5.8%; *n* = 118)	decreased appetite (5.9%; n = 7) cerebral infarction (5.1%; n = 6) leukopenia (5.1%; n = 6)	1. methylphenidate (15.4%; *n* = 490)	tic (6.7%; *n* = 33) decreased appetite (6.3%; n = 31) headache (5.9%; *n* = 29)
	2. etanercept (4.4%; *n* = 90)	injection site pain (24.4%; n = 22) condition aggravated (7.8%; n = 7) headache (5.6%; n = 5) injection site erythema (5.6%; n = 5) juvenile idiopathic arthritis (5.6%; n = 5)	2. atomoxetine (6.2%; *n* = 197)	suicidal ideation (18.3%; *n* = 36) aggression (14.2%; n = 28) nausea (7.6%; *n* = 15)
	3. insulin aspart (2.3%; n = 47)	blood glucose increased (63.8%; n = 30) product leakage (46.8%; n = 22) diabetic ketoacidosis (23.4%; n = 11)	3. allergens (2.2%; *n* = 71)	anaphylactic reaction (29.6%; n = 21) urticaria (29.6%; n = 21) dyspnoea (26.8%; n = 19)
13–17 years (*n* = 7251)	1. dienogest (4.7%; *n* = 187)	pulmonary embolism (21.9%; *n* = 41) deep vein thrombosis (15.0%; n = 28) pelvic thrombosis (9.6%; n = 18)	1. methylphenidate (10.1%; *n* = 315)	headache (5.4%; n = 17) suicide attempt (4.1%; *n* = 13) nausea (3.8%; *n* = 12)
	2. levonorgestrel (4.7%; n = 185)	drug ineffective (9.7%; n = 18) abdominal pain (7.6%; *n* = 14) headache (6.5%; n = 12)	2. isotretinoin (5.2%; *n* = 163)	blood creatine phosphokinase increased (12.9%; n = 21) depression (11.0%; n = 18) acne (6.1%; *n* = 10) headache (6.1%; n = 10)
	3. paracetamol (3.3%; *n* = 130)	suicide attempt (57.7%; *n* = 75) intentional overdose (35.4%; *n* = 46) vomiting (21.5%; n = 28)	3. atomoxetine (3.8%; *n* = 117)	suicidal ideation (11.1%; n = 13) nausea (10.3%; n = 12) aggression (9.4%; n = 11)

^a^ presented are the three drugs substances and their three related ADRs most frequently reported (PT-level) stratified by age and sex. Single drug substances and their combination products (if available) were summarized. Listed are only those ADRs for which more than two ADR reports were available. One ADR report may contain information about more than one drug substance and more than one ADR, therefore, the numbers of reported drug substances and ADRs may exceed that of the ADR reports

^b^ ADR reports for palivizumab were mostly related to a lack of efficacy

Table 5 presents the three drugs substances and their ADRs most frequently reported stratified by age and sex

### Reporting rates of the age- and sex-stratified analysis

Again, as shown for the reporting rates of the complete data set, the ranking differed if the age- and sex-specific exposure for these drugs was considered (Table 6). Antiepileptics (topiramate, levetiracetam and valproinic acid) ranked 1st to 3rd in children 2 months–1 year. Valproinic acid ranked 1st in children 2–3 years with a much lower reporting rate than observed for children 2 months-1 year. Antibiotics ranked 2nd and 3rd in children 2–3 years. In children 4–6 years, antiepileptics ranked 1st (oxcarbazepine) and 3rd (valproinic acid), methylphenidate ranked 2nd with a roughly 4 times greater reporting rate than for children 7–17 years. In contrast, etanercept ranked first in children 7–17 years. Reporting rates for ibuprofen were rather low for all age groups.

**Table 6:** Reporting rates of the five drug substances most frequently reported stratified by age and sex

Rank	2 month-1 year [number of ADR reports per 100,000 drug prescriptions] ^a^	2–3 years [number of ADR reports per 100,000 drug prescriptions] ^a^	4–6 years [number of ADR reports per 100,000 drug prescriptions] ^a^	7–12 years [number of ADR reports per 100,000 drug prescriptions] ^a^	13–17 years [number of ADR reports per 100,000 drug prescriptions] ^a^
1.	topiramat: 379.4	valproinic acid: 47.8	oxcarbazepine: 27.9	etanercept: 542.2	etanercept: 505.5
	female: 481.6	female: 48.9	female: 26.1	female: 503.9	female: 503.3
	male: 262.5	male: 40.6	male: 29.3	male: 575.6	male: 452.8
2.	levetiracetam: 250.7	amoxicillin: 1.3	methylphenidate: 22.8	insulin aspartat: 47.8	isotretinoin: 22.5
	female: 237.3	female: 1.0	female: 19.3	female: 46.6	female: 14.8
	male: 245.3	male: 1.4	male: 22.6	male: 48.9	male: 25.3
3.	valproinic acid: 161.0	cefaclor: 1.0	valproinic acid: 19.5	atomoxetine: 19.1	methylphenidate: 6.0
	female: 80.9	female: 0.8	female: 25.5	female: 14.9	female: 9.7
	male: 204.1	male: 1.2	male: 15.2	male: 19.4	male: 4.8
4.	palivizumab: 87.7 ^b^	salbutamol: 0.5	amoxicillin: 0.8	methylphenidate: 5.2	dienogest: 4.6
	female: 72.7 male: 88.4	female: 0.4 male: 0.5	female: 0.7 male: 1.0	female: 4.9 male: 5.0	female: 4.6 male: 0
5.	ibuprofen: 0.7	ibuprofen: 0.3	ibuprofen: 0.3	ibuprofen: 0.4	levonorgestrel: 2.8
	female: 0.5	female: 0.2	female: 0.2	female: 0.2	female: 2.8
	male: 0.7	male: 0.4	male: 0.4	male: 0.5	male: 0

^a^presented are the five drug substances most frequently reported. Single drug substances and their combination products (if available) were summarized

^b^ADR reports for palivizumab were mostly related to a lack of efficacy

Table 6 presents the ranking of the reporting rates (number of ADR reports/number of drug prescriptions) for the five drug substances most frequently reported in the age-stratified analysis of the complete data set (see Table 4)) as well as the reporting rates for females and males. The number of ADR reports for the respective drugs was determined for the time period 01/01/2009–28/02/2019, since substance-specific exposure data were only available for this time

As already described, in absolute numbers more ADR reports for methylphenidate referred to males than to females (Table 5). However, if related to sex-specific drug exposure the reporting rates for males and females 4–12 years were almost equal but differed with an almost two times higher reporting rate for females 13–17 years than for males (9.7 versus 4.8 ADR reports) (Table 6).

### Sex-stratified analysis of ADRs most frequently reported

Irrespective of the applied drug rather unspecific ADRs ranked first to third among females (“vomiting”, “nausea”, “dyspnoea”) and males (“urticaria”, “vomiting”, “dyspnoea”) (Table 7). However, for females “suicide attempt” ranked fourth and was more often reported compared to males (OR 4.3 [2.7–7.0]). Almost all of these ADR reports referred to females 13–17 years leading to “suicide attempt” as most reported condition in this age group (Supplementary Table 1). In contrast, “suicide attempt” ranked 13th with 2.9% (91/3119) of the ADR reports for males of the same age (Table 7). “Urticaria” (5.7%) and “dyspnoea” (5.5%) were most frequently reported for males 13–17 years.

**Table 7:** Sex-stratified analysis of ADRs most frequently reported

Rank	The ten ADRs reported most frequently in ADR reports referring to females (*n* = 9354) ^a^	The ten ADRs reported most frequently in ADR reports referring to males (*n* = 10,670) ^a^
1.	vomiting (6.2%; *n* = 581) OR: 1.3 [1.0–1.7]	urticaria (5.3%; *n* = 563) OR: 0.8 [0.6–1.0]
2.	nausea (4.7%; *n* = 443) OR 1.5 [1.1–2.1]	vomiting (4.8%; *n* = 515) OR: 1.3 [1.0–1.7]
3.	dyspnoea (4.2%; *n* = 390) OR: 0.9 [0.7–1.2]	dyspnoea (4.5%; *n* = 481) OR: 0.9 [0.7–1.2]
4.	suicide attempt (4.1%; *n* = 385) OR: 4.3 [2.7–7.0]	nausea (3.2%; *n* = 342) OR: 1.5 [1.1–2.1]
5.	urticaria (4.1%; *n* = 382) OR: 0.8 [0.6–1.0]	rash (3.2%; *n* = 340) OR: 0.9 [0.7–1.3]
6.	intentional overdose (3.3%; *n* = 312) OR: 5.0 [2.8–8.8]	anaphylactic reaction (3.2%; n = 339) OR: 0.6 [0.4–0.9]
7.	headache (3.2%; *n* = 303) OR: 1.2 [0.8–1.8]	foetal exposure during pregnancy (3.1%; *n* = 326) OR: 0.7 [0.5–1.1]
8.	tachycardia (3.2%; *n* = 301) OR: 1.3 [0.9–1.8]	pruritus (3.0%; *n* = 323) OR: 1.0 [0.7–1.4] pyrexia (3.0%; n = 323) OR: 0.9 [0.7–1.4]
9.	fatigue (3.2%; *n* = 300) OR: 1.3 [0.9–1.9]	seizure (2.8%; *n* = 297) OR: 1.0 [0.7–1.4]
10.	dizziness (3.1%; *n* = 386) 1.4 [0.9–2.0]	headache (2.7%; *n* = 285) OR: 1.2 [0.8–1.8]

OR = 1 no differences in sex-stratified analysis, OR > 1 ADR is more often reported for females, OR < 1 ADR is more often reported for males

^a^ one ADR report may contain information about more than one ADR, therefore, the number of reported ADRs exceeds that of the ADR reports

Table 7 shows the absolute and relative number of the ten most frequently reported ADRs on PT-level of the MedDRA terminology [25] stratified by sex and their calculated ORs with Bonferroni adjusted CI for females versus males

### Analyses of ADR reports referring to “off-label use”

In 3.5% (722/20,854) of the reports an off-label use was coded (Table 8). Differences were observed with regard to the drugs and ADRs reported most frequently in these reports compared to those reports not associated with “off-label use”. The three drugs reported most often as suspected in these ADR reports were aripiprazole 5.0% (36/722), levetiracetam 3.0% (22/722), and methylphenidate 2.6% (19/722).

**Table 8:** Analysis of ADR reports referring to “off-label use”

	ADR reports referring to “off-label use” (3.5%, *n* = 722*)
*The five drug substances most frequently reported* ^*a*^	
1.	aripiprazole (5.0%; 36/722)
2.	levetiracetam (3.0%; 22/722)
3.	methylphenidate (2.6%; 19/722)
4.	eculizumab (2.2%; 16/722)
5.	valproinic acid (2.2%; 16/722)
*The five ADRs most frequently reported (except PTs coding for off-label use)* ^*a*^	
1.	drug ineffective (4.0%; 29/722)
2.	seizure (3.9%; 28/722)
3.	fatigue (3.7%; 27/722)
4.	vomiting (3.5%; 25/722)
5.	nausea (2.9%; 21/722)

* in only 3.5% of the ADR reports an “off-label use” was coded. Hence, the ranking of the most frequently reported drug substances and ADRs in the remaining data set (complete data set excluding ADR reports referring to off-label use) remains the same as for the complete data set (see Table 1)

^a^ one ADR report may contain information about more than one drug substance and more than one ADR. Therefore, the number of reported drug substances and ADRs exceeds that of the ADR reports. Single drug substances and their combination products (if available) were summarized

If off-label use as per authorized age was considered, 2.7% (31/1149), 1.0% (3/286) and 1.0% (4/407) of the ADR reports referring to methylphenidate, risperidone and atomoxetine, respectively, were used off-label.

## Discussion

To the best of our knowledge, this is the first descriptive analysis of ADR reports originating from Germany with regard to the drugs and ADRs reported most frequently for children (0–17 years) performed in EudraVigilance. One major strength of our analysis is the consideration of the number of ADR reports in context with the number of drug prescriptions. In our study the ranking of the most frequently reported drugs in absolute numbers changed if the specific exposure of the drugs was taken into account. Differences in age. and sex-stratified analysis for the drugs and ADRs most frequently reported were observed, especially for methylphenidate. In absolute terms more ADR reports for methylphenidate referred to males. However, if drug exposure was considered, reporting rates for females aged 13–17 years were two times higher. The drugs most frequently involved in ADR reports associated with an “off-label use” differed from those reported in the whole dataset.

### ADRs reported most frequently

In our analysis, the five SOCs reported most frequently were in line with those observed in other ADR database studies [11, 18, 32–34] except for the SOC “injury, poisoning and procedural complications”. One reason for this finding could be that all of the referenced studies [11, 18, 32, 34] had a data lock point before the definition of an ADR was widened in 2012 (except for one study [18] where the data lock point was June 2013). Since 2012 the definition of an ADR is no longer confined to the use within the authorized indication but also includes ADRs related to “off-label use”, suicide/self-injury behavior and medication errors [20]. At least some of these ADRs which occurred with the use of the drug outside its authorized conditions are assigned to the SOC “injury, poisoning and procedural complications” [25]. Although these ADRs may have been reported before 2012, it seems likely that these ADRs were more often reported afterwards.

On the more detailed PT-level our results except for dyspnea were similar to those of an analysis of pediatric ADRs published in 2014 [18]. Further on, in a systematic review of studies performed in ADR databases, skin disorders like rash and urticaria were also most frequently observed [35]. Others discussed differences with regard to the skin physiology for children being responsible for a higher frequency of cutaneous ADRs compared to adults [34, 36].

### Drugs most often reported as suspected

Methylphenidate, ibuprofen, palivizumab, atomoxetine and etanercept were the drug substances most frequently reported as suspected in the complete data set. The ranking varied depending on age and sex, probably reflecting age- and sex-specific prevalent diseases and prescribing patterns, as also reported in other analyses [17, 34]. Additionally, the ranking changed if drug exposure was considered.

In our analysis, methylphenidate ranked first in the whole dataset and was over all ages 3.5 times more often reported for males compared to females. Methylphenidate and drugs for the treatment of attention deficit hyperactivity disorders (ADHD) ranked also 1st to 5th in a systematic analysis of ADR database studies [34]. Methylphenidate was the ADHD medication most often prescribed in Germany in the past [37]. In addition, regulatory actions and respective press releases may have stimulated ADR reports referring to ADHD medications [38, 39]. If our age-stratified analysis was related to the number of drug prescriptions, the reporting rates were roughly four-times higher for children 4–6 years (22.8 ADR reports) compared to the older ones (7–12 years: 5.2 ADR reports; 13–17 years: 6.0 ADR reports). Methylphenidate is approved for children older than 5 years and ranked third in our analysis of ADR reports referring to “off-label use” and first in our analysis of “off-label use” per authorized age. Furthermore, the ADRs most frequently reported differed between age groups. “Blood creatine phosphokinase increased” and “leukopenia” ranked first and second for methylphenidate in children 4–6 years. Both ADRs were more frequently reported for children 4–6 years compared to the older ones. Hence, based on our results caution is advised if younger children are treated with methylphenidate and, blood values should be monitored regularly in order to avoid these ADRs and their potential sequel.

With regard to the age- and sex-stratified analysis, the higher absolute number of ADR reports for methylphenidate referring to males may be caused by the higher prevalence of ADHD for males. Other studies estimated female/male ratios of 1:9 to 1:3 [40–43]. However, if related to the number of drug prescriptions, reporting rates were almost equal for males and females 4–12 years but almost two times higher for females 13–17 years compared to males. A more common occurrence for at least some ADRs (e.g. anxiety) for females treated with methylphenidate or ADHD medication compared to males was reported by others [44, 45]. Based on our findings female adolescents treated with methylphenidate seem to have a higher frequency of ADRs than males. In literature, a more frequent reporting of ADRs by adult females is discussed to likely impact on the number of ADR reports for females [45]. However, it is unknown if this may also apply for female children and adolescents with regard to ADR reporting or ADR communication to their parents or doctors. Nevertheless, a higher drug use by female adolescents (14–17 years) was observed in a German study about medication use in children and adolescents which may increase the risk of ADR occurrence [8]. Besides the exposure, ADRs in patients using drugs for the treatment of chronic diseases such as ADHD may be more likely to be recognized and reported due to a more closely monitoring of these patients than ADRs for the treatment of non-chronic diseases such as infections [46, 47].

Ibuprofen ranked second in our complete data set with the lowest reporting rate if compared to the other four drugs. Thus, the large absolute number seems to be caused by the frequent use of ibuprofen in Germany. In Germany, ibuprofen is available as prescription-only and over the counter (OTC) drug. Unfortunately, the amount of OTC use of ibuprofen was unknown. Although ibuprofen prescriptions are reimbursed in Germany for children until 12 years, OTC use cannot be excluded. Hence, the number of applications may be even higher. This would lead to an even lower reporting rate than the calculated one in our analysis. Ibuprofen ranked third in one study from North America (1 < 12 years) [48] and one from Italy [49] in a systematic ADR database analysis [35].

Palivizumab is a monoclonal antibody for the prevention of serious lower respiratory tract disease requiring hospitalization caused by respiratory syncytial virus (RSV). It is indicated for children at higher risk of RSV disease born at 35 weeks of gestation or less and younger than 6 months at the onset of the RSV season as well as for children younger than 2 years with bronchopulmonary dysplasia or congenital heart disease [50]. This may explain why in our analysis palivizumab was most frequently reported for children aged 2 months-1 year, only. The ADRs most frequently reported (40.1% respiratory syncytial virus infection, 16.1% respiratory syncytial virus bronchiolitis) rather suggested reporting of a lack of efficacy which was confirmed in an individual case analysis. If drug-specific exposure was taken into account it ranked only fourth in the age group 2 month – 1 year. In the systematic analysis of ADR databases [35] it was mentioned in one publication only (first rank) [51].

Atomoxetine is authorized for the treatment of ADHD in children aged 6 years and older. It ranked second and third for males 7–12 years and 13–17 years in our analysis. In Germany, it was the second most often prescribed ADHD medication until 2013 [37]. However, the considerably lower number of drug prescriptions compared to methylphenidate results in a higher reporting rate for atomoxetine compared to methylphenidate in our analysis, with a higher rate for males than for females aged 12–17 years. In the systematic review of ADR database analyses [35], atomoxetine was explicitly mentioned in one study only [32]. However, some of these studies listed ADHD medications as summarizing term [35]. Further on, a stimulated reporting for atomoxetine may have been caused by a Dear Doctor Letter informing about suicidality in children in 2005 [52]. This might also have influenced the observation that “suicidal ideation” was the condition most frequently reported for atomoxetine in our analysis. In addition, atomoxetine was authorized in Europe in 2004. An increased reporting of ADRs after a new drug has entered the market (so called *Weber effect*) was described by others [53].

Etanercept ranked third in children 7–17 years, however, it ranked first if drug-specific exposure was considered. Etanercept is authorized for the treatment of juvenile idiopathic arthritis and chronic severe plaque psoriasis [54]. Since these are severe diseases and etanercept is a biological, it may be assumed, that children taking etanercept are under regular medical surveillance. This would likely enhance the detection and reporting of ADRs. In the systematic review of ADR database analysis [35], etanercept was explicitly mentioned in one study only [18].

### ADRs and drugs reported most frequently stratified by age and sex

In our age-stratified analysis, the ADRs and drugs reported most frequently differed. “Foetal exposure during pregnancy”, “atrial septal defect” and “premature baby” were the conditions, and “drugs for the treatment of nervous system disorders” (e.g. venlafaxine, lamotrigine) were the drugs most often reported in children 0–1 month. The ADRs most frequently reported seemed to be associated with drug exposure during pregnancy. Other studies also observed, among others, prematurity, poor neonatal adaption and respiratory distress in neonates of women treated with antidepressants during pregnancy [55, 56]. Our calculated reporting rates for children 2 month-1 year were considerably higher for these drugs (e.g. for lamotrigine) compared to older children. These results should be interpreted with caution since we did not exclude ADR reports that reported drug exposure via the mother during pregnancy, which was the condition most frequently reported in this age group.

Irrespective of the applied drug, “vomiting” and “urticaria” ranked first for children 2–3 and 4–12 years. Hypersensitivity-like reactions (rash, urticaria etc.) were, among others, reported in association with amoxicillin and ibuprofen for children 2–3 and 4–6 years. This finding could likely reflect that acute common infections become more frequent for children aged 3–6 years like observed in a German investigation [57].

“Suicide attempt” was the condition independent of the applied drugs most often reported in children 13–17 years and more often reported for females than for males. However, with regard to the drugs and their ADRs most frequently reported, hormonal contraceptives and thromboembolic events ranked first for females 13–17 years. In contrast, for males of the same age, suicide associated with methylphenidate and atomoxetine was striking. In Germany, roughly one fifth (21.5%) of girls aged 14–17 years take hormonal contraceptives [8] which may impact on the higher number of ADR reports. Additionally, the high media attention in the past may have led to a more frequent reporting. However, if related to the number of drug prescriptions, reporting rates were rather low compared to the other drugs.

Finally, as also stated by others [17, 18] it should be noted that very young children may not be able to communicate specific ADRs, particularly those which are subjective. Instead unspecific and/or recognizable symptoms may dominate in these age groups and may lead to differences in the most frequently reported ADRs stratified by age. In addition, reporting of ADRs at least for younger children will generally involve the parents [18].

### Off-label use

The drugs substances most frequently reported in ADR reports referring to off- label use in our analysis differed from to the drugs most frequently reported in the whole dataset. Likewise they differed from the drugs most frequently used off-label according to a Germany survey [9]. In our analysis, drugs for the treatment of nervous system disorders were commonly reported. In contrast, the drugs most often used off-label in Germany were cardiovascular drugs, antineoplastic and immunmodulating agents and drugs for the treatment of sensory organs [9]. In this survey, drugs for the treatment of nervous system disorders played a rather minor role accounting for only 16% of off-label drugs. The potential to induce ADRs may differ between these drug classes leading to the observed differences between our study and the German survey. In addition, methodological differences in study designs have to be considered. As already mentioned, possibly not all ADR reports in which a drug was used off-label are designated with an off-label use, which may impact on our results.

With regard to the ten drugs most frequently reported overall (not restricted to off-label use) in our analysis, off-label use as per authorized age after exclusion of children aged 0–1 year was only observed for methylphenidate, risperidone and atomoxetine. All of these drugs are used for the treatment of nervous system disorders. Again, in the German study, drugs for the treatment of nervous system disorders played also a rather minor in the evaluation of drugs used off-label as per authorized age [9]. In that study, cardiovascular drugs, antineoplastic and immunmodulating agents and drugs for the treatment of musculoskeletal and connective tissue disorders were identified as the drugs most often used off-label per authorized age. It has to be considered that we only analyzed the proportion of off-label use per authorized age in the ten most frequently reported drugs of the whole dataset. However, the proportion of identified cases for drugs used off-label as per authorized age for these ten drugs was small. Off-label use as per authorized age (3.8%) ranked only fourth as reason for the off-label use after under-dosing (17.4%), over-dosing (4.6%) and indication (4.3%) in the Germany survey [9].

### Advantages and disadvantages of analysis using spontaneous reporting data

Strengths of our analysis include the coverage of a long period of time, the inclusion of a wide pediatric population in real life, the large number of ADR reports available for the analysis compared to many other national ADR database analyses [11, 15, 16, 33, 34] as well as the calculation of reporting rates based on the number of drug prescriptions.

One limitation of our analysis is the lack of exposure data specifically matching the individual in the ADR report. We addressed this limitation by using drug prescription data from the Central Research Institute for Ambulatory Health Care in Germany [29]. However, our analysis only presents the reporting rates of the five drugs most frequently reported. Other drugs which are not considered might have a higher reporting rate. In addition, the drug prescription data refer only to the reimbursed drugs prescribed in outpatients while our ADR reports may also be related to drugs used in hospitals.

Another limitation is the unknown amount of underreporting which may differ, among others, per age group, particular drug and nature of the ADR [58–60]. Due to the missing exact exposure data and the unknown extent of underreporting no incidences can be calculated based on the results of such analyses. Additionally, due to the large number of ADR reports, an individual case assessment of all ADR reports with regard to the causal association and the quality of the ADR report was not performed. However, all submitted ADR reports are suspected cases of ADRs and 77.0% of our random sample had an at least possible causal relationship which is in line with observations of other previous analyses [21, 22, 61]. Although, the completeness score of our random sample was 0.6 [0.6–0.7] and thus below the 0.8 expected for a well-documented report [30], it should be noted, that certain minimal criteria have to be present to allow the submission to EudraVigilance [20].

## Conclusions

In our analysis drugs for the treatment of ADHD were commonly reported. Thus, monitoring of children treated with ADHD drugs with regard to the occurrence of ADRs is recommended. In case of methylphenidate special attention should be paid to children aged 4–6 years and females aged 13–17 years. Further on, our analysis emphasizes that the number of ADR reports should not be interpreted as a self-standing figure but put in context with exposure data. In addition, sex- and age-specific analysis should be performed since these allow to identify subpopulations associated with a higher risk. Age- and sex-specific differences in diseases and drug exposures may also account for some of the observed differences in the number of ADR reports. With regard to off-label use, different drugs and ADRs seemed to be more relevant compared to the large group of ADR reports not associated with off-label use. In our analysis, the drugs most frequently suspected in the “off-label” ADR reports differed from those most frequently used off-label according to a German survey [9]. Possibly, the potential to cause ADRs may vary between drugs that are used off-label. Methodological differences and limitations between our study and the survey may account for the observed differences. In this respect, possibly not all ADR reports in which a drug was used off-label are designated with an off-label use. Further research is recommended in order to analyze whether certain drugs are associated with a higher risk of ADRs when used off-label.

## Supplementary Information

**Additional file 1.**

**Additional file 2.**

## Data Availability

The data which formed the basis for the current study are publicly accessible (public access: http://www.adrreports.eu/en/index.html). However, different levels of access are granted by the European Medicines Agency (EMA) for different stakeholders. The Federal Institute for Drugs and Medical Devices (BfArM) as a national competent authority is granted with the highest level of access since one of the core duties of the BfArM is to analyse EudraVigilance data in order to fulfill its pharmacovigilance obligations. A lower level of access is granted to researchers of other institutions, health care professionals and the public. Thus, the presented analysis may be performed on a higher level but not in that detail by other researchers. However, due to the tiered access granted by EMA it is not possible to make the data sets of the more detailed analysis publicly available.

## Abbreviations

ADHD: Attention Deficit Hyperactivity Disorders
ADR: adverse drug reaction
BfArM: Federal Institute for Drugs and Medical Devices
CI: Confidence Interval
EEA: European Economic Area
EMA: European Medicines Agency
EU: European Union
EVDAS: Data analysis system of EudraVigilance
MedDRA: Medical Dictionary for Regulatory Activities
NSAID: Non-Steroidal Anti-Inflammatory Drugs
OR: Odds Ratio
OTC drugs: Over-the-Counter drugs
PT: Preferred Term
RSV: Respiratory Syncytial Virus
SOC: System Organ Class
US: United States of America
WHO: World Health Organization
