# Supplementary material for: Descriptive analysis of adverse drug reaction reports in children and adolescents from Germany: frequently reported reactions and suspected drugs
Source: BMC Pharmacol Toxicol. 2021 Oct 7;22:56. doi: 10.1186/s40360-021-00520-y (PMC8499510; doi:10.1186/s40360-021-00520-y)
Supplement: Supplementary file 1 — Additional file 1. [file 40360_2021_520_MOESM1_ESM.docx]

**Suppelementary file 1) List of PT’s of the MedDRA terminology in order to identify the ADR reports referring to “off-label use”**

For the analysis of ADR reports referring to “off-label use” we combined the PTs

- “drug effective for unapproved indication”,
- “drug ineffective for unapproved indication”,
- “product use in unapproved indication”,
- “unintentional use for unapproved indication”,
- “off-label-use”,
- “product use in unapproved therapeutic environment”,
- “therapeutic product effective for unapproved indication”,
- “therapeutic product ineffective for unapproved indication”,
- “device use issue”,
- “product use issue”,
- “intentional device use issue”,
- “intentional product use issue”, and
- “off-label-use of device”.
