# Supplementary material for: Descriptive analysis of adverse drug reaction reports in children and adolescents from Germany: frequently reported reactions and suspected drugs
Source: BMC Pharmacol Toxicol. 2021 Oct 7;22:56. doi: 10.1186/s40360-021-00520-y (PMC8499510; doi:10.1186/s40360-021-00520-y)
Supplement: Supplementary file 2 — Additional file 2. [file 40360_2021_520_MOESM2_ESM.docx]

Supplementary Table 1) Age- and gender-stratified analysis of ADRs most frequently reported.

| age groups | the three ADRs reported most frequently in ADR reports referring to females (n= 9,354) ^1^ per age group | the three ADRs reported most frequently in ADR reports referring to males (n= 10,670) ^1^ per age group |
| --- | --- | --- |
| 0–1 month | n= 986  16.8% atrial septal defect (n= 166)  14.4% foetal exposure during pregnancy (n= 142)  13.0% premature baby (n= 128) | n= 1,329  17.2% foetal exposure during pregnancy (n= 229)  13.9% atrial septal defect (n= 185)  12.3% premature baby (n= 164) |
| 2 month–1 year | n= 898  6.9% vomiting (n= 62)  6.5% respiratory syncytial virus infection (n= 58)  6.3% foetal exposure during pregnancy (n= 57) | n= 1,212  7.2% foetal exposure during pregnancy (n= 87)  6.6% respiratory syncytial virus infection (n= 80)  5.6% vomiting (n= 68) |
| 2–3 years | n= 669  10.6% vomiting (n= 71)  8.7% accidental exposure to product by child (n= 58)  5.5% urticaria (n= 37) | n= 775  8.1% vomiting (n= 63)  7.9% accidental exposure to product by child (n= 61)  5.9% accidental overdose (n= 46) |
| 4–6 years | n= 787  8.3% vomiting (n= 65)  6.2% urticaria (n= 49)  5.3% pyrexia (n= 42) | n= 1,049  8.0% vomiting (n= 84)  7.3% urticaria (n= 77)  5.0% pruritus (n= 52)  5.0% pyrexia (n= 52)  5.0% rash (n= 52) |
| 7–12 years | n= 2,042  6.1% nausea (n= 125)  5.9% urticaria (n= 121)  5.9% vomiting (n= 120) | n= 3,186  7.3% urticaria (n= 234)  7.1% dyspnoea (n= 227)  5.6% anaphylactic reaction (n= 180) |
| 13–17 years | n= 3,972  9.5% suicide attempt (n= 376)  7.5% intentional overdose (n= 298)  6.6% nausea (n= 264) | n= 3,119  5.7% urticaria (n= 178)  5.5% dyspnoea (n= 172)  4.6% nausea (n= 145) |

^1^ one ADR report may contain information about more than one ADR, therefore, the number of reported ADRs exceeds that of the ADR reports.

Legend Supplementary Table 1):

Supplementary Table 1) presented the absolute and relative number of the three ADRs most frequently reported on PT-level of the MedDRA terminology stratified by age and gender.
